# Preoperative Urodynamics in Pelvic Organ Prolapse to Identify Postoperative Incontinence

**DOI:** 10.3390/jcm15145576

**Published:** 2026-07-16

**Authors:** Carolin Schröder, Michel Brodesser, Laura Tascón Padrón, Jakob F. Pantenburg, Lucia A. Otten, Eva K. Egger, Alexander Mustea, Dominique Koensgen

**Affiliations:** Department of Gynaecology and Gynaecological Oncology, University Hospital Bonn, Venusberg-Campus 1, 53127 Bonn, Germany

**Keywords:** latent stress urinary incontinence, urodynamics, pelvic organ prolapse

## Abstract

**Background/Objectives**: Stress urinary incontinence (SUI) can be masked by pelvic organ prolapse (POP), resulting in so-called latent SUI, and can also worsen postoperatively after POP surgery. Preoperative urodynamic (UD) testing can help identify such hidden dysfunctions, though its routine use and predictive value remain unclear. **Material and Methods**: This retrospective cohort study included women who underwent POP surgery between January 2019 and October 2025 and who underwent selective preoperative UD and postoperative follow-up (FU). The primary endpoint was to identify urodynamically latent SUI in women undergoing POP surgery, defined as clinical loss of urine during UD with prolapse reposition in patients without preoperatively diagnosed SUI. Secondary endpoints included evaluating de novo SUI rates, incontinence outcomes in women with preoperative UI, and the predictive value of UD for postoperative SUI in asymptomatic patients. SUI outcomes were assessed using validated questionnaires (International Consultation on Incontinence Questionnaire—Urinary Incontinence Short Form, German pelvic floor questionnaire), clinical stress testing, and anamnesis. **Results**: A total of 261 patients were included. Of those, 70 patients (26.8%) reported SUI, 16 (6.1%) urgency urinary incontinence, and 101 (38.7%) mixed urinary incontinence. Ninety-four patients (36%) received incontinence surgery. Latent SUI was identified in 13 patients (5%): of these, one underwent Burch colposuspension, and two underwent tension-free vaginal tape implantation, while the remaining 10 patients (76.9%) did not undergo incontinence surgery. At FU, 12 of 13 patients (92.3%) were continent; data were missing for one patient (7.7%). De novo SUI occurred in 6 patients (2.3%); these patients did not undergo incontinence surgery and did not show signs of latent SUI in UD. Preoperative UD demonstrated low sensitivity (0–44.4%) but moderate specificity (73.8–77.3%) for predicting postoperative SUI, with high negative predictive values (up to 98%), although these findings should be interpreted in light of the low event rates. **Conclusions**: In this selected cohort of women undergoing POP surgery, preoperative UD showed limited predictive value for postoperative SUI.

## 1. Introduction

Urinary incontinence (UI) in women is a prevalent and often underdiagnosed condition that significantly impacts quality of life. It affects up to half of all women during their lifetime, and its prevalence increases with age and parity. Pelvic organ prolapse (POP) occurs in up to 50% of parous women, and both conditions frequently coexist.

While stress urinary incontinence (SUI) and POP often present together, the clinical manifestation of incontinence can be masked by prolapse, resulting in a so-called latent SUI. This refers to SUI that becomes clinically apparent only after correction of the prolapse. Higher grades of POP are associated with lower rates of SUI [1,2]. Failure to recognise and address latent SUI before surgery can lead to overt or de novo postoperative urinary leakage, undermining surgical success and patient satisfaction.

Preoperative urodynamic (UD) testing provides objective measurements of lower urinary tract function and can unmask dysfunction. In the context of POP, UD with prolapse reduction techniques (e.g., pessary insertion or vaginal reposition) can simulate postoperative conditions and reveal latent SUI. Current guidelines acknowledge a potential role for prolapse reduction testing in women with advanced POP without symptoms of SUI; however, the routine use of urodynamics before POP repair remains controversial, and its impact on clinical outcomes remains uncertain [3]. Several studies have reported a strong influence on surgical decision-making and postoperative outcomes, whereas others raise concerns about cost-effectiveness and the lack of clinical benefit [4,5,6,7]. Despite ongoing debate, reliable identification of latent SUI and prediction of postoperative continence outcome remain important challenges in the management of women undergoing POP surgery. Edenfield et al. describe latent SUI as a common and clinically relevant condition, reporting postoperative rates of 40–50% in continent women with advanced POP and emphasising the importance of cough stress testing during prolapse reposition in preoperative counselling [8].

However, the clinical value of preoperative UD testing for identifying these patients and predicting postoperative continence outcomes remains uncertain. Therefore, the aim of this study was to evaluate the role of preoperative UD in women undergoing POP surgery. The primary endpoint was to identify urodynamically latent SUI in women scheduled for POP surgery. The secondary endpoints were the evaluation of de novo SUI rates, incontinence outcomes in women with preoperative UI, and the predictive value of UD for postoperative SUI in women without preoperative clinical UI. A better understanding of its diagnostic value could support individualised treatment and improve patient-centred outcomes.

## 2. Material and Methods

Study design and population: In this retrospective cohort study, patients who underwent POP surgery with preoperative UD between January 2019 and October 2025 at the Department of Gynaecology at the University Hospital Bonn were included. Patients without preoperative UD or neurological conditions, such as neurogenic bladder dysfunction or multiple sclerosis, were excluded.

Clinical assessment: Clinical data were collected retrospectively from the electronic patient file, including symptom-oriented urogynaecological history, examination, and transvaginal and perineal sonography before POP surgery and at the last follow-up (FU). Indications for POP repair were set when conservative treatment failed (pelvic floor muscle training, pessary treatment, local estrogen therapy, bladder training, and physiotherapy) or the symptom burden was high. The pelvic organ quantification system (POP-Q) was used to grade POP [9].

Clinical decision-making: Preoperative UD testing was performed in accordance with local clinical practice and at the treating urogynaecologist’s discretion. UD was primarily performed in women with UI symptoms, mixed or unclear lower urinary tract symptoms, voiding dysfunction, or when additional information was considered relevant for preoperative counselling and surgical planning. It was not routinely performed in all women undergoing POP surgery. The decision to perform a concomitant anti-incontinence procedure was based on a combination of clinical symptoms, physical examination, stress testing, UD findings, prolapse characteristics, and patient preference. Patients were counselled regarding the potential risk of postoperative SUI and the option of either concomitant continence surgery or a staged approach. In women without clear clinical SUI or in cases with uncertain risk of postoperative incontinence, POP repair was performed first, followed by postoperative reassessment of continence status. If bothersome SUI persisted or developed after POP correction, additional anti-incontinence surgery could subsequently be considered. Patients were informed that postoperative continence outcomes could not be predicted with certainty and that further treatment might be required in selected cases. The choice between Burch colposuspension (BC) and tension-free vaginal tape (TVT) implantation was made individually based on the planned prolapse procedure, anatomical findings, and shared decision-making with the patient; similarly, the choice of surgical approach (vaginal or robotic-assisted) was made. All surgical procedures were performed by one board-certified urogynecological surgeon at a tertiary referral centre.

Outcome measures: Subjective outcome was assessed using two validated questionnaires. The German pelvic floor questionnaire (GPFQ) covers four domains: bladder function, bowel function, prolapse symptoms, and sexual function. Each domain evaluates symptom-related distress and its impact on quality of life. Domain scores are summed, divided by the maximum possible score for each domain, and multiplied by 10. The total maximum score of the GPFQ is 40. Additionally, question 6 of the GPFQ was used to evaluate postoperative SUI (“How often do you lose urine when you cough or sneeze?”) [10]. The International Consultation on Incontinence Questionnaire—Urinary Incontinence Short Form (ICIQ-UI-SF) assesses the frequency and volume of urinary incontinence episodes as well as their overall impact on quality of life. Scores range from 0 to 21, with higher scores indicating more severe symptoms [11,12]. Questionnaires were self-completed by the patients before surgery and at postoperative FU visits. As this was a retrospective study based on routine clinical practice, neither patients nor treating physicians were blinded. As no universally accepted definition of postoperative SUI exists, both subjective and objective outcome measures were analysed. Patient-reported SUI symptoms assessed by anamnesis and validated questionnaires were considered clinically most relevant.

Incontinence definitions: SUI was defined according to the International Urogynecological Association (IUGA) and International Continence Society (ICS) terminology as the complaint of involuntary loss of urine on effort or physical exertion, or on sneezing or coughing [13]. The severity of SUI symptoms was classified according to the Stamey classification [14]. Urodynamic SUI was defined as involuntary leakage of urine during increased abdominal pressure in the absence of a detrusor contraction during UD. Urgency urinary incontinence (UUI) involves involuntary leakage with a sudden, hard-to-defer urge to void and is part of the overactive bladder (OAB) symptom complex. Mixed urinary incontinence (MUI) combines SUI and UUI symptoms. Latent SUI is defined as any SUI that becomes apparent after surgical and temporary correction (e.g., pessary or swab) of POP in patients without anamnestic or clinical symptoms of SUI preoperatively [9]. Intrinsic sphincter insufficiency (ISD) was defined as maximal urethral closure pressure (MUCP) ≤ 20 cm H_2_O [15,16]. De novo SUI was defined as new-onset SUI occurring after POP repair in patients without preoperative anamnestic, clinical or UD evidence of SUI or latent SUI.

Urodynamic (UD) testing: UD was performed with prolapse repositioning. The method used to reduce prolapse during UD testing was not standardised. Two different reduction techniques, pessary use and vaginal swab repositioning, were used in accordance with routine clinical practice. A fluid-filled catheter system was used for multi-channel UD testing, including cystometry, urethral pressure profile, and electromyography. Measurements were taken after bladder filling with sterile sodium chloride at a rate of 20 mL/min until the patient’s maximum bladder capacity was reached or at least 350 mL. Bladder volume at the first urge to urinate, maximum bladder capacity, MUCP, and functional urethral length (each measured at rest and under the Valsalva manoeuvre), as well as residual urine volume, were measured. Additionally, detrusor overactivity, negative pressure transmission, and clinical loss of urine during UD were documented. During bladder filling, the stress test (coughing) was repeated at bladder volumes of 50 mL, 100 mL, 150 mL, 200 mL, and so on, with the urethral catheter in situ. Repeat stress testing after catheter removal was not routinely performed.

Follow-up: As this was a retrospective study, FU intervals were not standardised and reflected routine clinical practice. Clinical examination, symptom-oriented history, and, when available, validated questionnaires were used to assess postoperative continence outcomes. For the present analysis, only preoperative data and data from the last available FU visit were evaluated.

Statistical analysis: The statistical analysis was performed using SPSS 29.0.2.0 (IBM Corp., 2023, IBM SPSS Statistics for Windows, Version 29.0. Armonk, NY, USA: IBM Corp.). Continuous variables were compared between the latent SUI group and the control group (preoperatively continent patients without latent SUI) using the Mann–Whitney U test, while categorical variables were analysed using Fisher’s exact test. Sensitivity, specificity, and positive and negative predictive values were calculated for four postoperative SUI definitions to assess the diagnostic performance of preoperative UD. Patients who underwent incontinence procedures were excluded from this analysis to avoid bias introduced by the interventions. Given the small number of latent SUI and de novo SUI cases, only exploratory univariable analyses were performed. A *p* < 0.05 was considered statistically significant. As this was a retrospective study based on routine clinical practice, questionnaire completion and FU assessments were not available for all patients. Therefore, the number of patients included in each analysis varied according to data availability. No imputation of missing data was performed. The study was approved by the ethics committee of the University of Bonn with the reference number 2025-415-BO. This study was registered in the German Clinical Trials Register (DRKS) under the number DRKS00038815 on 23 December 2025, after patient recruitment had already started, because of its retrospective study design. The retrospective registration did not influence patient management, data collection, or outcome assessment.

## 3. Results

**Figure 1:**
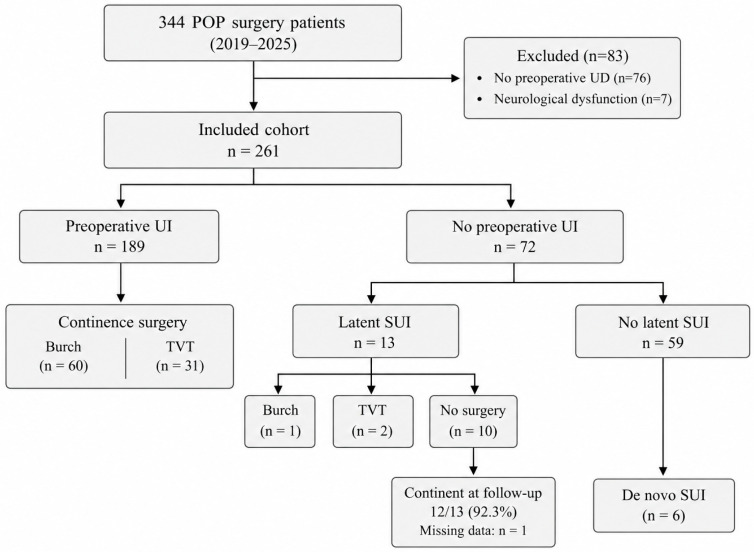
Study cohort and continence outcomes.

Between January 2019 and October 2025, 344 patients underwent POP repair at the Department of Gynaecology at the University Hospital Bonn. A total of 76 patients were excluded because no preoperative UD had been performed; 7 were excluded due to neurogenic bladder dysfunction or other neurological conditions. A total of 261 patients were included in the final analysis (Figure 1). The median age was 63 years (range 31–89). The mean body mass index (BMI) was 26 kg/m^2^ (range 18–42).

Forty-two patients (16%) had a prior POP or incontinence procedure. The mean duration of POP symptoms at the first consultation in our department was 2.5 months (range 0–24). Preoperatively, 187 patients (71.6%) reported UI symptoms, including SUI in 70 patients (26.8%), UUI in 16 (6.1%), and MUI in 101 (38.7%). Conservative therapy had already been initiated on an outpatient basis in 178 patients (68.2%). A total of 127 patients (48.7%) underwent a vaginal POP procedure, and in 134 patients (51.3%), the procedure was performed by robotic-assisted laparoscopy. Concomitant incontinence procedures were performed in 94 patients (36%). Patients’ characteristics and surgical data are summarised in Table 1 and Table 2.

Figure 1 illustrates the study cohort, patient selection process, and continence outcomes following POP surgery, including the distribution of preoperative UI, latent SUI, concomitant continence procedures, and postoperative continence status. For transparency and to illustrate the selection process, baseline characteristics of the excluded patients without preoperative UD were compared with those of the included cohort (Table 3).

## 4. Primary Endpoint

Latent SUI: Preoperative UD revealed no evidence of SUI in 144 patients (55.2%). Of the 117 patients (44.8%) with urodynamic signs or risk factors for SUI, 74 (28.4%) demonstrated urinary leakage during UD, 17 (6.5%) had ISD (MUCP ≤ 20 cm H_2_O), and 26 (10%) exhibited both findings.

Thirteen patients (5% of the total cohort) demonstrated urinary leakage during UD despite the absence of anamnestic SUI symptoms, corresponding to latent SUI. Of these, one patient underwent BC and two received TVT insertion, while the majority (10 patients, 76.9%) did not undergo any concomitant incontinence surgery. Follow-up reflected routine clinical practice and was therefore heterogeneous in duration. For the total cohort, FU was after a median time of 3 months (mean 7 months, range 0–56). Continence data were available for 12 of these patients, and all were continent. FU information was missing for one patient.

Patients with latent SUI (*n* = 13) had a median FU of 4 months (mean 7 months; range 2–22 months). Postoperative continent patients had a median FU of 3 months (mean 8 months, range 1–56).

Among the 13 patients with latent SUI, the mean age was 64.7 (median 64 years, range 38–84). Nine patients (69.2%) had no prior urogynecological surgery, whereas 4 (30.8%) had undergone previous POP surgery. Importantly, none of the patients had a history of previous continence surgery. Most patients presented with advanced anterior compartment prolapse, with 53.8% having POP-Q stage III anterior prolapse. Apical prolapse was predominantly stage II (61.5%), and posterior prolapse was stage II in 53.8% of patients.

Univariable analysis: Exploratory univariable analyses did not identify significant associations between latent SUI and age, BMI, POP stage, previous POP surgery, or type of POP surgery. A significant association was observed between latent SUI and concomitant continence procedures (*p* = 0.002), reflecting the clinical decision to perform anti-incontinence surgery in selected patients with latent SUI.

To characterise latent SUI, patients without preoperative anamnestic UI (*n* = 72) were analysed. Overall, 13 patients showed a latent SUI, compared to 59 continent controls without latent SUI (Table 4).

No statistically significant differences were observed between groups in any urodynamic parameter, including bladder capacity, MUCP at rest or stress, functional urethral length, ISD, or negative pressure transmission.

## 5. Secondary Endpoints

De novo SUI rates: Until last FU, six patients (2.3% of the total cohort) developed de novo SUI, defined as new-onset SUI in the absence of preoperative symptoms, UD evidence of SUI or latent SUI, and without concomitant incontinence surgery. In total, 12 patients (4.6%) developed de novo UI, including nine with SUI and three with MUI. In the remaining six patients with de novo UI, preoperative UD had already shown signs of SUI or ISD. Two of these patients had received concomitant BC, while four had not undergone any anti-incontinence procedure.

In a subanalysis, patients with de novo SUI (*n* = 6) had a median FU of 2 months (mean 3 months, range 1–6).

FU of ≥12 months was available for 85 patients of the total cohort, with a median follow-up of 12 months (mean 16 months, range 12–56 months). In a sensitivity analysis restricted to patients with a follow-up of ≥12 months and without preoperative incontinence or incontinence surgery (*n* = 18), no cases of de novo SUI were observed, and no additional cases were identified beyond the first 12 postoperative months in this subgroup.

Of the 76 patients initially excluded from the main analysis because no preoperative UD was performed, 3 patients developed de novo SUI (3.9%).

Incontinence outcomes in women with preoperative UI: Overall, 94 of 261 patients (36%) underwent a concomitant anti-incontinence procedure (61 BC [23.4%], 33 TVT [12.6%]).

Most of these patients (94.7%) presented with urethral hypermobility, and 11.7% had a positive cough stress test. Only one patient had a positive cough test without urethral hypermobility.

At FU, objective urine leakage during the stress test was observed in 10 patients (3.8%). In total, 65 patients (24.9%) reported SUI in daily life. According to the ICIQ-UI-SF, 110 patients (42.1%) were classified as incontinent (score ≥ 1), which was consistent with the GPFQ results (116 patients, 44.4%). Worsening of pre-existing UI was rare (3 patients, 1.6%), whereas 112 patients (42.9%) reported improvement in UI symptoms.

POP-Q postoperative: The results for postoperative POP-Q are shown in Table 5. Urethral hypermobility postoperatively was rotatory in 63 patients (24.1%), vertical in 14 patients (5.4%), and combined in 1 patient (0.8%). In 163 patients (62.5%), urethral hypermobility was absent postoperatively.

Predictive value for postoperative SUI in women without preoperative clinical UI: Because concomitant continence procedures may substantially alter postoperative outcomes, patients who underwent such procedures were excluded from the predictive analyses, and *n* = 68 patients were included in this subanalysis. Postoperative continence was assessed using four outcome definitions: ICIQ-UI-SF, GPFQ question six, clinical stress test, and anamnestic SUI (Table 6).

Across all outcome measures, preoperative UD demonstrated consistently low sensitivity (0–44.4%), but moderate specificity (73.8–77.3%). The positive predictive value was low, particularly for clinically assessed and anamnestic SUI (0–5.9%), whereas questionnaire-based outcomes showed moderate positive predictive values (53.5–61.5%). In contrast, the negative predictive value was consistently high, reaching 98% for the clinical stress test and 90% for anamnestic SUI. These diagnostic performance measures should be interpreted with caution, as the small number of latent and de novo SUI events may yield unstable estimates of sensitivity, specificity, and predictive values.

## 6. Discussion

In this large cohort of women undergoing POP repair, the overall predictive value of preoperative UD for postoperative SUI was limited. Several randomised studies and systematic reviews show that routine UD testing before SUI surgery in women with clinically proven SUI does not improve postoperative continence rates or reliably predict postoperative SUI [17,18,19]. For patients with more complex findings (e.g., mixed incontinence, previous surgery, prolapse), UD may provide additional information. However, the sensitivity and specificity of UD for predicting postoperative SUI are not clearly defined [20].

The observed latent SUI rate in our cohort was 5%, which is lower than the 23–50% reported in several previous studies [8,21,22]. Potential explanations include differences in patient selection, prolapse severity, prolapse-reduction techniques during UD, definitions of latent SUI, and local clinical practice regarding referrals for UD evaluation. Different prolapse reduction techniques have been shown to yield varying detection rates of latent SUI [23]. Because prolapse reduction was not standardised and the specific method was not documented, underdetection of latent SUI cannot be excluded and may partly explain the comparatively low latent SUI rate observed in our study. These methodological differences may have contributed to a systematic underdetection of latent SUI compared with previous studies and should be considered when interpreting the comparatively low prevalence observed in our cohort.

Our findings align with those of the FINPOP study by Karjalainen et al., a large prospective Finnish cohort evaluating UI outcomes after POP surgery [24]. FINPOP demonstrated that de novo SUI after prolapse repair is relatively uncommon and that routine prophylactic anti-incontinence procedures do not provide a clear overall benefit for all patients. Importantly, FINPOP showed that many women remain continent postoperatively despite preoperative risk factors, and that overtreatment may expose patients to unnecessary complications without improving patient-reported outcomes [24]. Similarly, in our cohort, postoperative de novo SUI occurred in only 2.3% of patients without preoperative anamnestic or urodynamic evidence of SUI or latent SUI, which was lower than in previously reported studies [25,26]. Continence data were available for 12 of 13 patients with latent SUI, and all patients with available FU remained continent, supporting a conservative and individualised approach rather than routine combined incontinence surgery.

When evaluating the diagnostic value of preoperative UD in predicting postoperative SUI among preoperatively continent women without concomitant incontinence surgery, UD consistently showed low sensitivity across all postoperative outcome definitions (0–44.4%), but moderate and stable specificity (73.8–77.3%). The positive predictive value was low, particularly for clinically assessed or anamnestic SUI, indicating that a positive UD finding poorly identifies women who will develop postoperative UI. In contrast, the negative predictive value was high, especially for the postoperative stress test (98%) and anamnestic SUI (90%). However, this should be interpreted with caution, as NPV is strongly dependent on disease prevalence. Given the very low incidence of de novo SUI, the high NPV may largely reflect the low postoperative event rate rather than a strong ability of preoperative UD to exclude future SUI. Nevertheless, our findings are in line with van der Ploeg et al., who demonstrated the limited predictive value of demonstrable SUI and the lack of added value of UD over basic stress testing [27]. Further studies of prolapse surgery report a high negative predictive value of up to 98% for the absence of postoperative SUI if no SUI was detectable in the preoperative UD [5]. These values resemble those reported by Toptas et al., who found a sensitivity of 60.7% and a higher specificity (up to 88%) for predicting postoperative leakage based on preoperative UD [4]. These findings are in line with the FINPOP study, which emphasised the limited ability of preoperative diagnostics to accurately predict postoperative SUI [24].

Among women with pre-existing UI, postoperative worsening was rare, whereas symptom improvement was frequent and occurred even in the absence of concomitant incontinence surgery. Given the very low number of worsening events and the strong influence of concomitant procedures, no meaningful association between preoperative UD findings and postoperative symptom changes could be established in this subgroup. The relatively high proportion of concomitant continence procedures reflects local clinical practice in a tertiary referral centre and may have influenced postoperative continence outcomes.

Overall, the influence of preoperative UD on clinical decision-making in our cohort was modest. Importantly, postoperative continence outcomes remained favourable regardless of whether UD-guided concomitant surgery was performed, further supporting the conclusions drawn from FINPOP that routine preoperative UD and prophylactic incontinence surgery offer limited additional benefit.

This study has several limitations. First, its retrospective, single-centre design inherently limits causal inference and generalisability. Incomplete FU and missing data may have introduced attrition bias. Because the mechanism of missingness was not formally assessed, we cannot rule out the possibility that patients with missing outcome data differed systematically from those with complete FU. For example, women experiencing postoperative urinary symptoms may have been either more likely to attend follow-up because of persistent complaints or, conversely, less likely to return to our tertiary referral centre and instead seek care elsewhere. Consequently, the reported rates of postoperative and de novo SUI may have been over- or underestimated. The relatively short median follow-up of 3 months (for the total cohort) may have also resulted in an underestimation of delayed de novo SUI, as continence status may evolve beyond the early postoperative period. Although no cases of de novo SUI were observed among the small subgroup of patients with follow-up ≥12 months, this subgroup was underpowered. Therefore, the absence of events should not be interpreted as evidence that delayed de novo SUI does not occur. Furthermore, only women who underwent preoperative UD were included. In routine clinical practice, UD was performed selectively at the treating urogynaecologist’s discretion, predominantly in women with UI symptoms or more complex clinical presentations. Consequently, the study cohort represents a selected population and may not fully reflect unselected women undergoing POP surgery, introducing substantial selection bias; furthermore, confounding by indication cannot be excluded. As demonstrated by the comparison with patients who did not undergo preoperative UD (Table 3), women undergoing UD were younger, more frequently had urinary incontinence, and were substantially more likely to receive concomitant continence surgery, whereas patients without UD more often presented with advanced apical prolapse. Therefore, the diagnostic performance of preoperative UD observed in this study may not be reproducible in an unselected POP population.

Second, the relatively small number of latent SUI (*n* = 13) and de novo SUI (*n* = 6) events limits the robustness of subgroup analyses and predictive performance measures. Sensitivity, specificity, and predictive values should therefore be interpreted with caution, as these estimates may be unstable and partly reflect the low postoperative event rate. Given the small number of latent SUI and de novo SUI events, the study may have been underpowered to detect clinically relevant differences in urodynamic parameters between groups. Stress testing during UD was performed with the urethral catheter in situ and repeat stress testing after catheter removal was not routinely performed. Because catheter removal may increase sensitivity to detecting stress urinary leakage, this is another reason latent SUI may have been underestimated in our study.

Third, the relatively high proportion of concomitant continence procedures may have influenced postoperative continence outcomes. The decision to perform concomitant anti-incontinence surgery was individualised and based on clinical symptoms, physical examination, urodynamic findings, surgical approach, and patient preference. This introduces treatment allocation bias and complicates the interpretation of the independent predictive value of preoperative UD.

Fourth, follow-up duration was relatively short and heterogeneous, reflecting routine clinical practice. Consequently, longer-term outcomes, particularly POP recurrence and long-term continence, may have been underestimated.

Finally, postoperative continence was assessed using multiple subjective and objective definitions, potentially introducing outcome heterogeneity. Additionally, prolapse reduction during urodynamic testing was not standardised, and different techniques may vary in their ability to detect latent SUI [23]. Future prospective multicentre studies with standardised testing protocols and longer FU are needed to further clarify the role of preoperative UD in women undergoing POP surgery.

## 7. Conclusions

Although positive preoperative UD findings showed limited predictive value, negative findings were associated with favourable postoperative continence outcomes in this selected cohort. In this context, a conservative and individualised approach to preoperative UD and concomitant anti-incontinence procedures appears appropriate. However, these findings should not be generalised to unselected populations of women undergoing POP surgery.

## Figures and Tables

**Table 1:** Patients’ characteristics.

	*n* = 261
Age, years, median (range)	63 (31–89)
BMI, kg/m^2^, mean ± SD (range)	26 ± 4 (18–42)
Vaginal delivery (≥1), n (%)	253 (97)
Number of vaginal deliveries, median (range)	2 (0–8)
Nulligravida, n (%)	2 (0.8)
Menopausal status distribution, n (%) Premenopausal Perimenopausal Postmenopausal Missing data	24 (9.2) 10 (3.8) 225 (86.2) 2 (0.8)
ASA classification ^a^, n (%) Grade I Grade II Grade III Grade IV	48 (18.4) 182 (69.7) 30 (11.5) 1 (0.4)
Relevant Comorbidities, n (%) Previous or current breast cancer Diabetes type I or II Psychological (e.g., depression)	19 (7.3) 17 (6.5) 15 (5.7)
Previous urogynecological operations, n (%) Previous POP surgery Previous SUI surgery Previous POP + SUI surgery	33 (12.6) 4 (1.5) 5 (1.9)
Duration of POP symptoms, months, mean ± SD (range)	2.5 ± 3.3 (0–24)
Previous conservative treatment, n (%) None Unimodal Multimodal (≥2 treatment modalities) Missing data	84 (32.2) 102 (39.1) 58 (22.2) 17 (6.5)
Type of incontinence, n (%) none SUI UUI MUI Missing data	72 (27.6) 70 (26.8) 16 (6.1) 101 (38.7) 2 (0.8)
Stamey classification of SUI preoperative ^b^, n (%) Grade I Grade II Grade III Missing data	105 (40.2) 59 (22.6) 4 (1.5) 3 (1.1)
POP-Q anterior compartment, n (%) none Grade I Grade II (−1 to +1) Grade III Grade IV	11 (4.2) 22 (8.4) 149 (57.1) 78 (29.9) 1 (0.4)
POP-Q apical compartment, n (%) None Grade I Grade II (−1 to +1) Grade III Grade IV	26 (10) 29 (11.1) 142 (54.4) 60 (23) 4 (1.5)
POP-Q posterior compartment, n (%) None Grade I Grade II (−1 to +1) Grade III Grade IV	113 (43.3) 76 (29.1) 64 (24.5) 7 (2.7) 1 (0.4)
Urethral hypermobility, n (%) None Rotatoric Vertical Combined Missing data	36 (13.8) 186 (71.3) 8 (3.1) 11 (4.2) 20 (7.7)

^a^ American Society of Anesthesiologists (I: healthy, II: mild systemic disease, III: severe systemic disease). ^b^ Stamey classification [14]: urine incontinence during: I: coughing, sneezing, II: running, climbing stairs, III: without physical activity; SD = standard deviation.

**Table 2:** Surgical data.

	*n* = 261
Vaginal POP surgery, n (%) Anterior/posterior colporrhaphy ± sacrospinous vault suspension MESH-augmented transvaginal prolapse repair ± colporrhaphy Others	109 (41.7) 16 (6.1) 2 (0.8)
Robotic-assisted POP surgery, n (%) Sacrocervicopexy Sacrocolpopexy Hysteropexy Others	114 (43.7) 16 (6.1) 3 (1.1) 1 (0.4)
Concomitant incontinence procedure, n (%) Burch colposuspension Tension-free vaginal tape None	61 (23.4) 33 (12.6) 167 (64)
Length of inpatient hospital stay, median (range)	4 (1–19)

**Table 3:** Comparison of patients with (*n* = 261) and without (*n* = 76) preoperative urodynamic.

	With Preoperative Urodynamic *n* = 261	Without Preoperative Urodynamic *n* = 76
Age, years, median (range)	63 (31–89)	70 (31–88)
BMI, kg/m^2^, mean ± SD (range)	26 ± 4 (18–42)	26 ± 5 (10–37)
Type of incontinence, n (%) none SUI UUI MUI Missing data	72 (27.6) 70 (26.8) 16 (6.1) 101 (38.7) 2 (0.8)	38 (50) 10 (13.2) 11 (14.5) 17 (22.4) 0
POP-Q anterior compartment, n (%) none Grade I Grade II (−1 to +1) Grade III Grade IV	11 (4.2) 22 (8.4) 149 (57.1) 78 (29.9) 1 (0.4)	13 (17.1) 4 (5.3) 24 (31.6) 30 (39.5) 3 (3.9)
POP-Q apical compartment, n (%) None Grade I Grade II (−1 to +1) Grade III Grade IV	26 (10) 29 (11.1) 142 (54.4) 60 (23) 4 (1.5)	8 (10.5) 5 (6.6) 23 (30.3) 25 (32.9) 15 (19.7)
POP-Q posterior compartment, n (%) None Grade I Grade II (−1 to +1) Grade III Grade IV Missing data	113 (43.3) 76 (29.1) 64 (24.5) 7 (2.7) 1 (0.4) 0	34 (44.7) 17 (22.4) 17 (22.4) 4 (5.3) 3 (3.9) 1 (1.3)
Vaginal POP surgery, n (%) Anterior/posterior colporrhaphy ± sacrospinous vault suspension MESH-augmented transvaginal prolapse repair ± colporrhaphy Others	109 (41.7) 16 (6.1) 2 (0.8)	38 (50.0) 13 (17.1) 2 (2.6)
Robotic-assisted POP surgery, n (%) Sacrocervicopexy Sacrocolpopexy Hysteropexy Others	114 (43.7) 16 (6.1) 3 (1.1) 1 (0.4)	16 (21.1) 3 (3.9) 4 (5.3) 0
Concomitant incontinence procedure, n (%) Burch colposuspension Tension-free vaginal tape None	61 (23.4) 33 (12.6) 167 (64)	0 1 (1.3) 75 (98.7)

**Table 4:** Analysis of UD parameters in patients with latent SUI vs. without latent SUI.

Patients Without Preoperative UI (*n* = 72)	Latent SUI *n* = 13	Control Group Without Latent SUI *n* = 59	*p*-Value
First urge to urinate, mL, mean ± SD (range)	223 ± 116 (82–450)	204 ± 79 (58–412)	*p* = 0.884
Maximum bladder capacity, mL, mean ± SD (range)	358 ± 84 (215–467)	350 ± 81 (131–591)	*p* = 0.533
Detrusor overactivity, n (%)	0	0	N.A.
Intrinsic sphincter insufficiency, n (%)	3 (23.1)	8 (13.6)	*p* = 0.083 *
Negative pressure transmission, n (%)	10 (76.9)	23 (39)	*p* = 0.115 *
Maximum urethral closure pressure (rest), cmH_2_O, mean ± SD (range)	50 ± 24 (9–94)	63 ± 32 (15–145)	*p* = 0.106
Maximum urethral closure pressure (stress), cmH_2_O, mean ± SD (range)	44 ± 29 (9–115)	56 ± 41 (3–228)	*p* = 0.094
Functional urethral length (rest), mm, mean ± SD (range)	13 ± 6 (5–22)	19 ± 9 (4–49)	*p* = 0.082
Functional urethral length (stress), mm, mean ± SD (range)	15 ± 9 (6–34)	19 ± 11 (2–44)	*p* = 0.915
Residual urine volume, n (%)	0	0	N.A.

SD = standard deviation.; N.A. = not available; * Fisher’s Exact test, others Mann–Whitney U-test.

**Table 5:** Postoperative stages of pelvic organ prolapse.

Postoperative POP-Q	POP-Q Stage 0 *n* (%)	POP-Q Stage I *n* (%)	POP-Q Stage II *n* (%)	Missing Data *n* (%)
Anterior compartment	213 (81.6)	32 (12.3)	8 (3.1)	8 (3.1)
Middle compartment	250 (95.8)	2 (0.8)	4 (1.5)	5 (1.9)
Posterior compartment	200 (76.6)	53 (20.3)	3 (1.1)	5 (1.9)

POP-Q stages III and IV were not observed in any patient. No exploratory testing was performed due to the small number of patients with POP-Q stage ≥ II.

**Table 6:** Predictive value of preoperative urodynamics for postoperative SUI.

Postoperative Marker	Sensitivity	Specificity	Positive Predictive Value	Negative Predictive Value
ICIQ-UI-SF (*n* = 41)	42.1%	77.3%	61.5%	60.7%
GPFQ (question 6) (*n* = 45)	44.4%	74.1%	53.5%	66.7%
Stress test (*n* = 66)	0%	73.8%	0%	98%
Anamnestic SUI symptoms (*n* = 67)	16.7%	73.8%	5.9%	90%

## Data Availability

The dataset and materials used in this study are available from the corresponding author upon reasonable request.

## Abbreviations

ASA	American Society of Anesthesiologists
BC	Burch colposuspension
BMI	Body mass index
DRKS	German Clinical Trials Register
FU	Follow-up
GPFQ	German pelvic floor Questionnaire
ISD	Intrinsic sphincter deficiency
ICIQ-UI-SF	International Consultation on Incontinence Questionnaire Urinary Incontinence Short Form
MUI	Mixed urinary incontinence
MUCP	Maximum urethral closure pressure
OAB	Overactive bladder
POP	Pelvic organ prolapse
POP-Q	Pelvic organ prolapse quantification system
SD	Standard deviation
SUI	Stress urinary incontinence
TVT	Tension-free vaginal tape
UD	Urodynamics/Urodynamic testing
UI	Urinary incontinence
UUI	Urgency urinary incontinence
